# Diagnostic performance of broad-range PCR in bacterial peritonitis

**DOI:** 10.3389/fcimb.2025.1645965

**Published:** 2025-10-01

**Authors:** María Miguélez Sánchez, Martine P. Bos, Lauren Remijas, Tom Lancee, Robin van Houdt, Andries E. Budding

**Affiliations:** 1 inBiome B. V., Amsterdam, Netherlands; 2 Department of Medical Microbiology &Infection Control, Amsterdam UMC, location Vrije Universiteit Amsterdam, Amsterdam, Netherlands

**Keywords:** peritonitis, molecular diagnosis, broad range PCR assay, ascites fluid infection, PMN (polymorphonuclear leucocyte), molecular diagnosis and epidemiology of enteric infections

## Abstract

**Objective:**

Bacterial peritonitis (BP) is a serious complication commonly associated with cirrhosis and ascites, often leading to high mortality rates. Although these effects could be reduced with timely and appropriate antibiotics, traditional BP diagnosis relies on culture, often delaying targeted treatment. Therefore, the use of fast molecular assays holds the potential to enhance laboratory diagnosis. In this study, we assessed the diagnostic performaance of Molecular Culture ID, a broad PCR-based assay targeting the 16S-23S interspace rDNA region in the scope of BP diagnosis.

**Methods:**

The residual material from 247 peritoneal fluid samples submitted for routine diagnostics was analyzed using Molecular Culture ID and compared alongside the standard of care (SOC) results.

**Results:**

Sample positivity and species identification outcomes of Molecular Culture ID were compared to those of SOC. Molecular Culture ID yielded 1.6x more positive samples than SOC. Percent positive agreement (PPA) between Molecular Culture ID and SOC at the sample level was 90.1% (IC 95%, 81.0% to 95.1%), and negative percent agreement (NPA) was 70.5% (IC 95%, 63.3% to 76.7%). At the species level, the PPA was 75.2% (95% CI 67.2% to 81.8%). Molecular Culture ID yielded 289 extra bacterial identifications, mainly anaerobic species. High leukocyte counts, indicative of infection, were concordant with Molecular Culture ID positivity.

**Conclusion:**

Molecular Culture ID demonstrated enhanced BP diagnostic capabilities compared to SOC, with higher positivity rates, more comprehensive species identification for difficult to culture species and a high correlation with leukocyte counts.

## Introduction

Bacterial peritonitis (BP) is an infection of the peritoneal cavity, often involving ascitic fluid, which can emerge as a severe complication in patients with underlying health issues. Peritonitis is classified as primary, secondary, or tertiary (Principles and practice of infectious diseases, 2019). Primary, often monomicrobial, affects children and cirrhotic patients (Zhang and Faust, 2021). Secondary, usually polymicrobial, results from gastrointestinal or genitourinary breaches and accounts for 80–90% of cases (Principles and practice of infectious diseases, 2019; Sartelli et al., 2024). Tertiary arises from unresolved infections, often involving immune dysfunction or resistant pathogens (Li et al., 2022). Mortality rates from this condition increase over time, ranging from 30% in earlier stages to 60% beyond 12 months (Arvaniti et al., 2010). Given that there is a critical window of BP treatment before multi-system organ failure or bloodstream infection, a rapid and adequate diagnosis is key to maximizing the chances of a positive outcome. The current diagnostic hallmark for BP is the absolute polymorphonuclear neutrophil (PMN) count in peritoneal fluid. Counts over 250 neutrophil cells/mm3 are used as indicators of subjacent infection (Dever and Sheikh, 2015). Those patients are empirically treated with broad-spectrum antibiotics, since pathogen’s identification and antibiotic sensitivity testing via culture can extend up to 5 to 7 days, leading to prolonged antibiotics exposure and an increased risk of resistance. Additionally, the mortality risk can escalate in a matter of hours if not given the right antibiotic treatment (Arabi et al., 2012), emphasizing the need for faster and more specific diagnostics. Thus, alternative diagnostic methods have been explored to supplement PMN counts. New molecular techniques focus on reducing the time to diagnosis, while increasing the sensitivity and specificity, and may provide fast pathogen identification (Such et al., 2002; Bruns et al., 2009). These techniques are particularly relevant for 20% of the cases which report high PMN and negative culture (neutrocytic ascites) (Runyon and Hoefs, 1984), or when PMN counts are < 250 cells/mm3 and positive for culture (bacterascites) (Runyon et al., 1988). Recently described molecular methods are based on 16S rRNA gene amplification combined with sequencing or high-resolution melt analysis for pathogen identification (Hardick et al., 2012; Goelz et al., 2021; Aehling et al., 2024). These techniques demonstrated higher sensitivity compared to traditional culture, reporting significantly more positive samples. However, 16S workflows are not high-throughput enough for clinical use and fail to provide an accurate outcome in low-biomass specimens, like ascites fluid (Yang et al., 2024). High-resolution melt analysis workflows are well suited for a clinical setting but present limitations when analyzing polymicrobial samples (Hardick et al., 2012).

In this study, we evaluate the application of Molecular Culture ID (MC-ID), a broad PCR-based molecular assay, for BP diagnosis. This technology targets the 16S and 23S polymorphic interspace (IS) rDNA with phylum-specific fluorescently labelled primers, allowing for bacterial identification at the species level. All bacterial species contain at least one IS region in their chromosome. However, many species contain multiple alleles of this region, exhibiting variation in nucleotide length. This characteristic allows fingerprint-like species identification, as IS profiles are conserved within the same species but highly diverse between species. Amplifying these fragments with phylum-specific fluorescent primers adds information to resolve potential overlaps in species identification. The amplified IS fragments are matched to bacterial species using software linked to a database of known IS region lengths (Budding et al., 2016). In addition, MC-ID contains an internal control that is used to benchmark amplification efficiency. The assay was previously tested with success in the diagnosis of infections occurring in various sterile body sites, and complex sample types, demonstrating the suitability for detecting a broad range of species (Budding et al., 2010; Budding et al., 2016; Bos et al., 2023; Bos et al., 2025). The MC-ID workflow can be completed in about 5 h, yielding a much faster turnaround time than culture and mNGS.

Given these features, we propose that MC-ID may provide a more comprehensive approach to BP diagnosis compared to traditional microbiological analysis. To test this notion, we retrospectively evaluated the performance of both methods using 247 peritoneal effusion samples.

## Materials and methods

### Sample collection

Residual material of 247 consecutive peritoneal effusion samples from 209 patients that were sent for routine diagnostics was collected between 2013 and 2019 at the Department of Medical Microbiology of Amsterdam UMC, location VU Medical Centre (VUmc) at Amsterdam, The Netherlands. The VUmc’s Medical Ethical Review Board ruled that this study was not subject to the Dutch Medical Research Involving Human Subjects Act (WMO) since subjects did not undergo any therapeutic or diagnostic procedures.

### Standard of care diagnostics

Upon arrival, part of the sample was aliquoted and stored at -80°C for later analysis with MC-ID. The remaining material was pelleted and resuspended in supernatant. This suspension was used for Gram stain; leucocyte count and culture. Cultures were grown on chocolate and blood agar (aerobic/anaerobic) and in Brain Heart Infusion broth. Secondary plates were inoculated from broth after 5 days if no growth appeared on primary plates. Colonies were identified via MALDI-TOF VITEK-MS (bioMérieux, Marcy-l’Étoile, France). Culture loads were categorized as negative, 0.1 (positive only on secondary plates), 1 (limited growth, only in the first streaking segment), 2 (intermediate growth into the second streaking segment, 10–100 colonies), or 3 (abundant growth, present in all three streaking segments, >100 colonies). Leucocyte counts were reported as none, low (1 or 2 per viewing field), medium (2–10 per viewing field), or high (>10 per viewing field).

### Molecular Culture ID

Molecular Culture ID (inBiome B. V., Amsterdam, The Netherlands, CE-IVD, IVDR 2024) assay was carried out according to the manufacturer’s instructions for use (IFU). Briefly, 50–200 µl of sample was mixed with 200 µl AL buffer (Qiagen) and 20 µl proteinase K, vortexed, spun down, and incubated at 56 °C and 1400 rpm for 1 hour to lyse material. One ml EasyMag lysis buffer (bioMérieux) was added before DNA extraction using the Specific A Protocol on the automated EMAG^®^ system (bioMérieux), with DNA eluted in 70 µl. Two PCRs were performed on 10 µl DNA each: one targeting Firmicutes, Actinobacteria, Fusobacteria, Verrucomicrobia, and Bacteroidetes; the other targeting Proteobacteria, an internal amplification control, and human DNA. PCR products were combined and analyzed for amplicon size and fluorescence (RFU) using capillary electrophoresis (ABI3500, ThermoFisher). Cutoffs for positivity were used as described in MC-ID IFU. Additionally, negative controls were incorporated in each DNA isolation round of 23 samples. Species identification was performed by the software platform antoni (inBiome B. V., Amsterdam, The Netherlands). In brief, the software performs the following steps: preprocessing, fragment calling, nucleotide size mapping, fragment classification, matching algorithm, abundance calculation and quality control. The preprocessing step takes as input ‘.fsa’ files containing the fluorescence signal and timepoints detection from the capillary electrophoresis. First, it corrects fluorescence artifacts and removes the noise signal, followed by the fragment calling steps. The amplified DNA fragments are mapped to nucleotide lengths using the size marker reagent with known nucleotide lengths as reference. Then follows a classification of the fragments as bacterial, human, and internal control. The matching algorithm couples the bacterial fragments (amplicon length and fluorescence) to bacterial species present in the reference database using a probabilistic approach. These outcomes are displayed in antoni alongside abundances per species based on fluorescence intensities (categorized as high, medium and low) and results of the quality control, indicating which samples require reinjection or dilution.

For uncertain outcomes, i.e. fragments for which the algorithm did not find a confident match within the database, the MC-ID PCR products were sequenced for further species identification.

### Sequencing

MC-ID PCR products were sequenced on a MinION device (Oxford Nanopore Technologies). PCR products were diluted 1000× and re-amplified using the two MC-ID primer sets. Barcoded libraries were prepared (SQK-LSK109, EXP-NBD196 kits) and sequenced on R9 flow cells. Reads containing MC-ID forward and reverse primers were extracted from the FASTA files and used to generate consensus sequences. These sequences were classified via BLAST (NCBI nr/nt and WGS databases), retaining hits with >95% query coverage and identity (for detailed results, see **Supplementary Table S4**).

### Data analysis

For the concordance analysis, only culture results positive for bacteria within phyla detectable by the MC-ID primer sets were considered SOC-positive. MC-ID results compared to culture outcome are reported as positive percent agreement (PPA) and negative percent agreement (NPA) since culture is regarded as an imperfect reference standard (U.S. Department of Health and Human Services Food and Drug Administration, 2007).

Extraction of the oxygen requirements and Gram stain characteristics was performed using the search engine *BacDive* (BacDive) and a custom script to query the species name output from MC-ID. Statistical analyses were performed using the Fisher exact test for which a p-value < 0.05 was considered statistically significant.

## Results

### Sample characteristics

**Figure 1:**
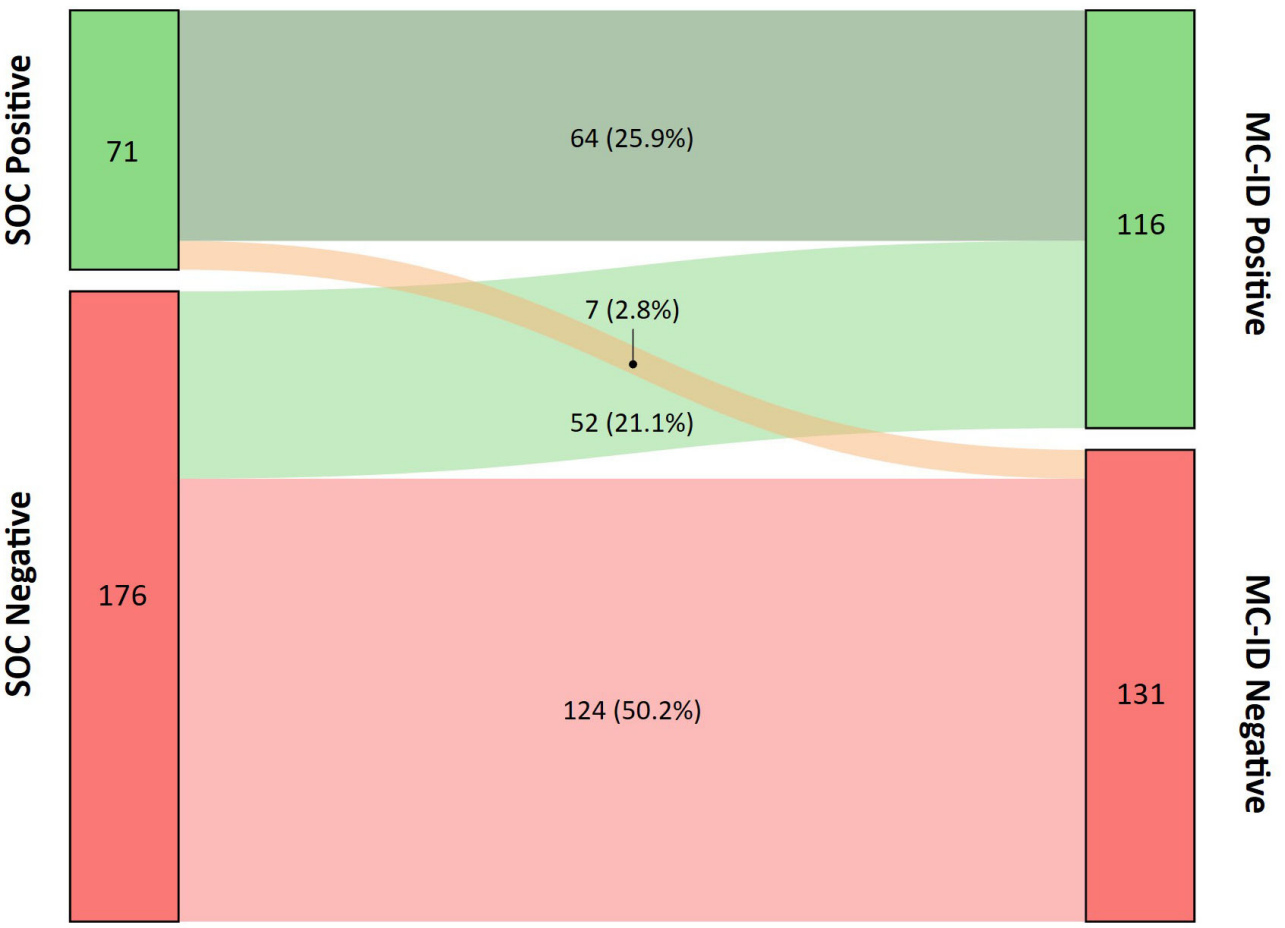
Sankey diagram presenting the distribution of sample positivity between SOC and Molecular Culture ID (MC-ID). The nodes represent the positive and negative samples by SOC on the left and MC-ID on the right, respectively. The widths of the links correspond to the proportion of samples which transition from one node to another. Percentages reflect the relative distribution based on the total sample count (n =247).

Positivity rates of 247 peritoneal effusion samples from 209 patients were compared between the standard of care (SOC) and MC-ID. The average age of participants was 59.5 years old, and the gender distribution was 59.1% male and 40.9% female. Of the 247 samples, 71 were positive in SOC (28.7%), whereas 116 samples (47%) were positive in MC-ID, with 64 samples concordant (**Table 1**). Two samples, where SOC identified species outside of MC-ID primer coverage (*Candida albicans* and *Mycoplasma hominis*), were considered as concordant negative. PPA between MC-ID and SOC at the sample level was 90.1% (IC 95%, 81.0% to 95.1%), NPA was 70.5% (IC 95%, 63.3% to 76.7%). MC-ID yielded 1.6x more positive samples than SOC (**Figure 1**).

**Table 1:** Sample level results from SOC and MC-ID.

Sample level outcome	SOC	MC-ID	Concordant
Positive	71	116	64
Monomicrobial	38	35	
Polymicrobial	33	81	
Negative	176	131	124
Total	247	247	188

### Species identifications by SOC and MC-ID

Of the 133 species identifications by SOC, 100 were concordant with MC-ID. PPA at the species level was 75.2% (95% CI 67.2% to 81.8%) (**Table 2**). The species most often detected by both methods were common BP pathogens such as *Escherichia coli, Enterococcus faecalis* and *Enterococcus faecium* (Ding et al., 2019). Most samples with discrepant species identifications were polymicrobial for both methods (79%). For all but one of those samples, MC-ID found at least one species concordant with SOC (**Supplementary Table S1**).

**Table 2:** Summary of species identified by SOC and their concordance with MC-ID.

Species	SOC	MC-ID	Concordant
Enterococci	40	52	31
*Enterococcus faecalis*	19	26^2^	15
*Enterococcus faecium*	20	25^3^	16
*Enterococcus gallinarum*	1	1	0
Enterobacteriaceae	36	53	32
*Citrobacter freundii*	1	5	1
*Citrobacter koseri*	1	1	0
*Escherichia coli*	18	28	16
*Enterobacter cloacae*	6	6^1^	6
*Klebsiella aerogenes*	1	1	1
*Klebsiella oxytoca*	2	2^1^	2
*Klebsiella pneumoniae*	4	7^1^	3
*Proteus vulgaris^a^*	3	3^1^	3
Staphylococci	24	21	13
Coagulase Negative *Staphylococci ^b^*	2	1^1^	1
*Staphylococcus aureus*	6	3	2
*Staphylococcus capitis*	2	2	1
*Staphylococcus epidermidis*	8	12^4^	7
*Staphylococcus haemolyticus*	3	3	2
*Staphylococcus pasteuri*	1	0	0
*Staphylococcus warneri*	2	0	0
Streptococci	8	39	6
*Streptococcus agalactiae*	1	2^2^	1
*Streptococcus anginosus*	3	4^2^	3
*Streptococcus bovis* group	1	16^1^	0
*Streptococcus pneumoniae/mitis* group	2	16^2^	1
*Streptococcus pyogenes*	1	1	1
Other	25	43	18
*Achromobacter xylosoxidans*	1	1	1
*Acinetobacter* species	1	0	0
*Actinomyces neuii*	1	1^1^	1
*Bacillus* species	1	0	0
*Bacteroides fragilis*	2	10^3^	2
*Clostridium perfringens*	1	4	1
*Corynebacterium tuberculostearicum*	1	0	0
*Cutibacterium acnes*	2	6	1
Fecal microbiota^c^	4	4	4
Other	25	45	18
*Lactobacillus (para)gasseri*	1	4	1
*Lactobacillus* species	1	4	1
*Neisseria subflava*	1	1	0
*Morganella morganii*	2	0	0
*Pseudomonas aeruginosa*	1	2^1^	1
*Sphingomonas paucimobilis*	1	1	1
*Stenotrophomonas maltophilia*	4	5^1^	4
TOTAL	133	208	100(75,2%)

a For one sample, SOC identified the species as *Proteus vulgaris* while MC-ID identified the species as *Proteus penneri*. This identification was backed up with sequencing. Due to difficulty in distinguishing *Proteus* species in culture, this was considered concordant (Rhoads et al., 2025).

b The identification by MC-ID was *Staphylococcus epidermidis*, which we considered concordant to Coagulase negative *Staphylococci*.

c Identifications by MC-ID were considered concordant with fecal microbiota, containing identifications of bacteria from the genus *Bacteroides, Alistipes* and *Prevotella*, among others.

The superscript for MC-ID identifications indicates the number of identifications obtained through sequencing. Species identified by MC-ID only are listed separately in **Supplementary Table S2**.

**Figure 2:**
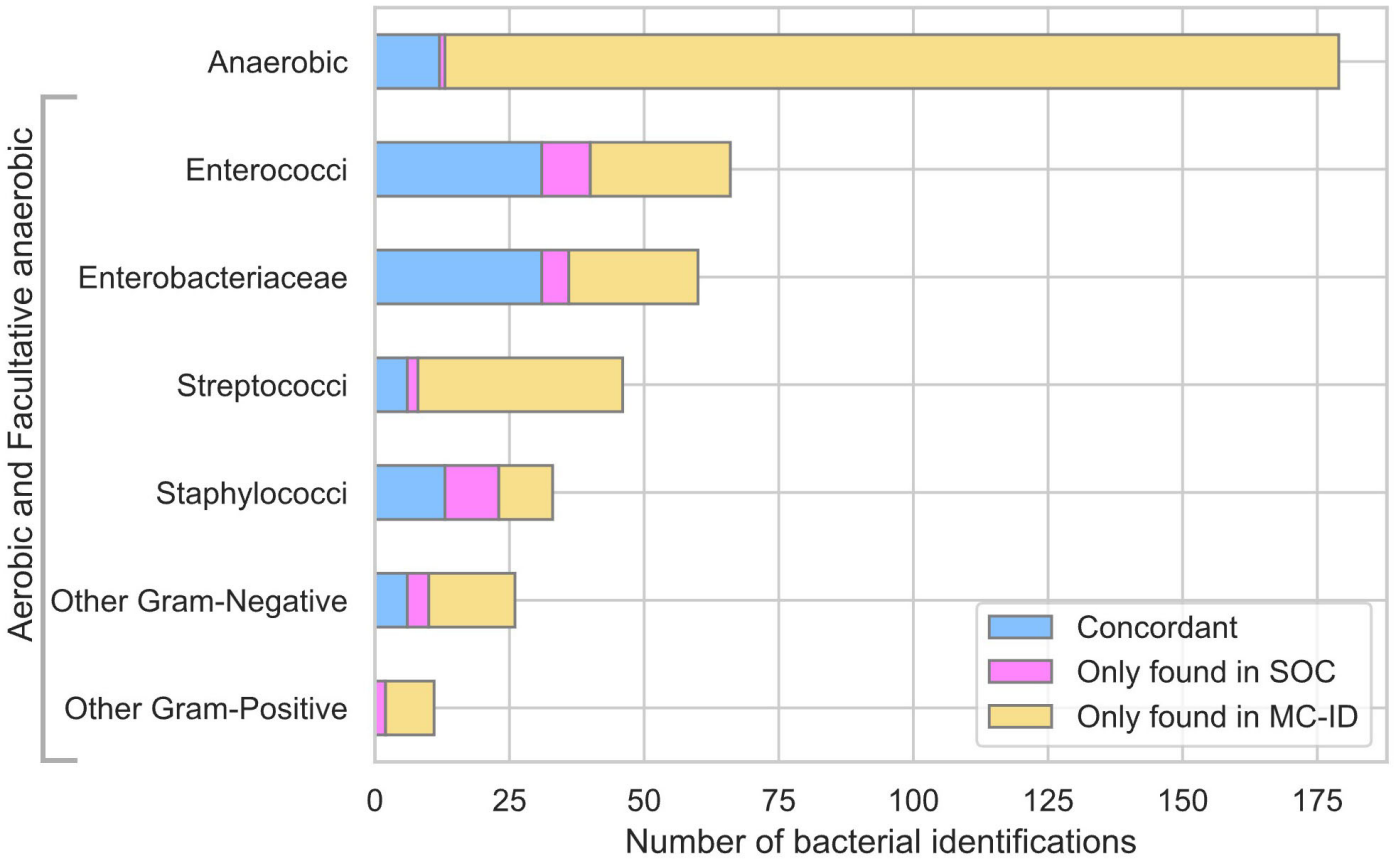
Bar plot of bacterial identifications by SOC and Molecular Culture ID (MC-ID) classified by main family, microscopy (Gram-positive/Gram-negative) and oxygen requirements (Anaerobic/Aerobic and Facultative anaerobic).

MC-ID yielded 289 extra bacterial identifications, representing a 2.9-fold increase in bacterial detections compared to SOC (**Supplementary Table S2**). Of the 289 extra identifications, 56.6% correspond to anaerobic species (**Figure 2**). The species with the highest number of additional identifications belong to typical gut microbiota, such as *Alistipes* spp. (18 x), *Bacteroides dorei* (12x) and *Sutterella wadsworthensis* (12x). Additionally, MC-ID identified higher numbers of common BP pathogens compared to SOC (*Streptococcus* spp. (36x), *Enterococcus* spp. (25x) and *Escherichia coli* (12x)).

### Evaluation of discrepant detections

In seven cases, SOC output was reported as positive, and MC-ID was negative (**Figure 1**). All these discrepant samples yielded low or very low bacterial loads (0.1-1). Three of the discrepant samples contained potential skin contaminants such as *Staphylococcus haemolyticus, Staphylococcus warneri*, or *Staphylococcus pasteuri.* Two samples were positive for *Enterococcus faecium* and *Staphylococcus aureus*, respectively. In two samples with low-load *Cutibacterium acnes* and *Escherichia coli*, MC-ID found signals corresponding to these bacteria. However, since the signal yield fell below the positive threshold (described in MC-ID IFU), it was considered noise and therefore reported as MC-ID negative.

Discrepant samples that were MC-ID-positive and SOC-negative represented 44.8% of the total MC-ID-positive sample set (**Figure 1**). These samples exhibited levels of bacterial detections comparable to those samples positive for both methods (**Supplementary Table S3**).

### Leukocyte counts in relation to MC-ID outcome

**Figure 3:**
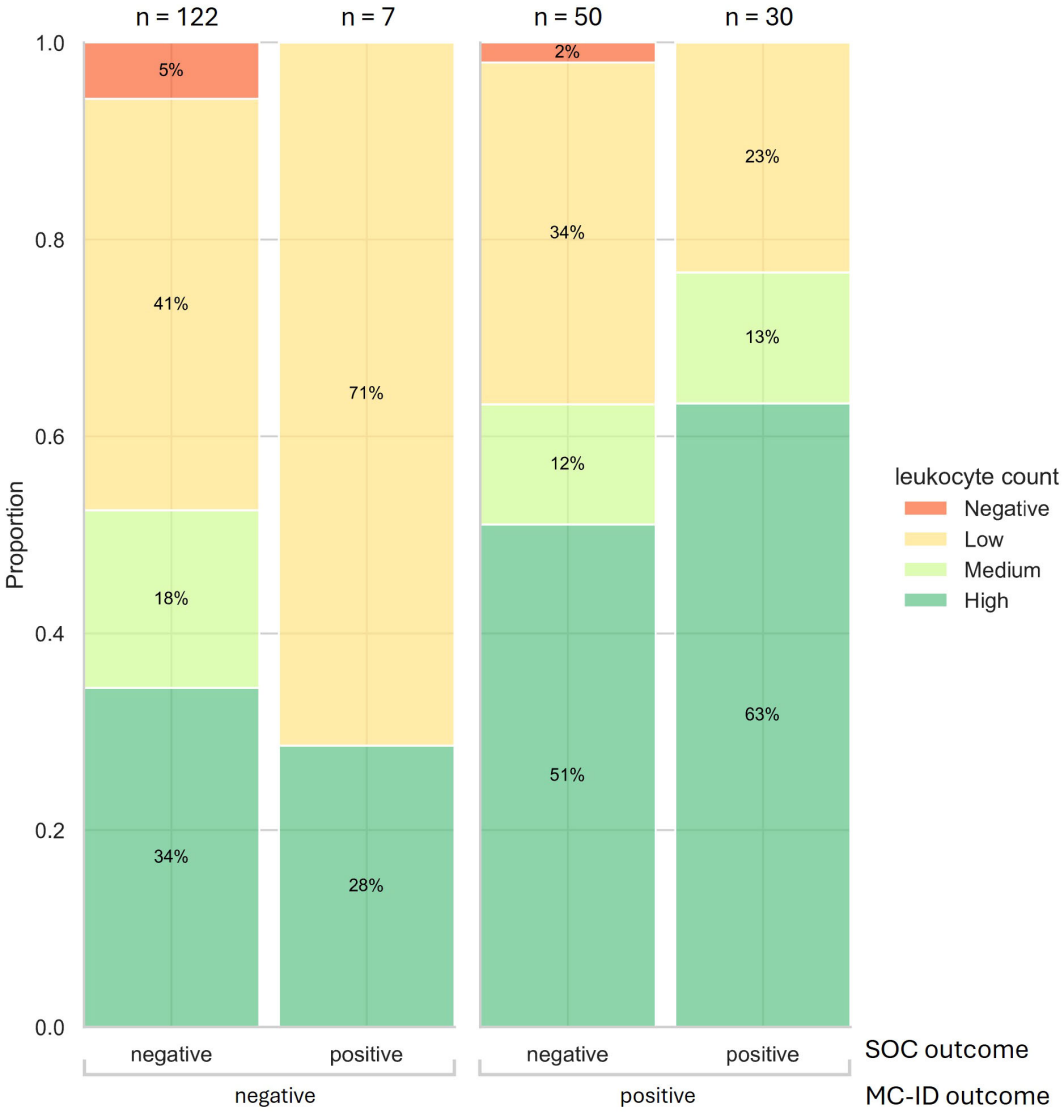
Stacked cumulative bar plot displaying the leukocyte count outcome per sample and its positive/negative outcome from SOC and MC-ID (only including samples with a leukocyte count result, n = 209).

Leukocyte counts (Negative, Low, Medium, High) were compared across outcomes from SOC bacterial culture and MC-ID to assess their association with infection detection. Samples without leukocyte count reports (n=38) were excluded from this analysis. Among all samples with an annotated result (n = 209), high to medium leukocyte counts were significantly more prevalent in MC-ID-positive samples (44/80) than in MC-ID-negative samples (44/129) (Fisher’s exact test, p = 0.004). Contrarily, high leukocyte levels were not significantly different between SOC-positive (21/37) and SOC-negative (67/172) outcomes (Fisher test, p-value > 0.05) (**Figure 3**). This finding indicates a correlation between positive MC-ID outcome and high leukocyte counts. Notably, one third of the 94 SOC-negative samples reporting high or medium leukocyte count (neutrocytic ascites) were MC-ID-positive. Anaerobic species were found in most of these samples (**Supplementary Table S3**).

## Discussion

In this study, we evaluate the performance of MC-ID for the diagnosis of bacterial peritonitis in a set of 247 peritoneal effusions. The positivity rate of MC-ID (47%) was significantly greater (Fisher test, p-value < 0.05) than that of SOC (28.7%) (**Figure 1**). This finding aligns with other studies reporting higher positivity levels for molecular techniques as compared to SOC (Bruns et al., 2009; Jafri et al., 2019; Aehling et al., 2024). MC-ID showed a detection PPA of 90.1% (IC 95%, 81.0% to 95.1%) and NPA of 70.5% (IC 95%, 63.3% to 76.7%) at the sample level (**Table 1**), revealing a high concordance between both techniques.

At the species level, the PPA was 75,2% (95% CI 67.2% to 81.8%) (**Table 2**), presenting a high concordance between the species found by SOC and MC-ID, especially for common BP pathogens (Mazuski et al., 2017; Ding et al., 2019). However, discrepancies where MC-ID did not identify the species found by SOC should be taken into closer consideration.

SOC yielded 33 extra bacterial identifications. The majority of these identifications were found in polymicrobial samples, where MC-ID typically identified at least one concordant bacterium (**Supplementary Table S1**). Notably, two-thirds of these bacterial identifications yielded low or very low bacterial loads (0.1-1). *Staphylococcus aureus*, a pathogen related to dialysis (Principles and practice of infectious diseases, 2019), was discordant in four cases (**Supplementary Table S1**). Possibly, this discrepancy is caused by the difficulty of lysing the thick cell wall to release DNA (Chapaval et al., 2008). Another potential cause is the different sample input volume used for both methods (SOC: 1 ml, MC-ID: 10-30 μL effectively present in the PCR), perhaps why a number of low-load SOC detections are missed by MC-ID (**Supplementary Table S1**).

MC-ID yielded 289 extra bacterial identifications, which, summed with the concordant identifications, represent a threefold increase in detected species compared to SOC (**Supplementary Table S2**). These identifications were sustained with sequencing (**Supplementary Table S4**). MC-ID identified high numbers of common BP pathogens, among which *Streptococcus* spp. (36x) was the least represented in SOC (**Figure 2**). This finding is concordant with studies comparing molecular techniques with culture, in which Streptococci identifications are largely underestimated due to facultative anaerobic culturing requirements (Bogiel et al., 2021; Bayala et al., 2024). Anaerobic bacteria were underrepresented in SOC, accounting for over half (56%) of the extra MC-ID identifications (**Figure 2**). The detected anaerobic species primarily correspond to common gut microbiota species, which are a typical cause of peritonitis due to bacterial translocation or perforation of the gastrointestinal cavity (Chetty et al., 2023). *Bacteroides* spp., the most common anaerobic pathogens in intrabdominal infections (Mazuski et al., 2017), accounted for the highest number of additional identifications (43x) (**Supplementary Table S2**). *Alistipes* spp. and *Sutterella wadsworthensis* were the second most found anaerobes, a notable finding given that these species are rarely reported as a causative agent of peritonitis due to their strict anaerobic growth requirements. A number of studies point out that molecular techniques have been able to identify this genus with greater reliability (Cobo et al., 2020; Fernández Vecilla et al., 2023). Moreover, anaerobe species were predominant in samples with a negative culture result and high leukocyte counts (**Supplementary Table S3**). These findings highlight the enhanced capabilities of MC-ID in the identification of novel anaerobic pathogens, which otherwise might be overlooked by culture.

Positive MC-ID results were strongly correlated with high leukocyte counts, regardless of SOC outcomes (**Figure 3**). In one third of SOC-negative cases with high and medium leukocyte counts, MC-ID identified a potential causative agent of infection, possibly explainable by anaerobe species predominance. These findings demonstrate MC-ID’s ability to identify infections that align with host inflammatory responses. Moreover, it underscores the superior sensitivity of MC-ID compared to SOC and highlights its potential to improve the detection of clinically relevant infections.

However, this study also presented certain limitations. SOC methods lacked anaerobic recovery qualities. The use of alternative media, such as Thioglycolate Broth Culture, could have provided superior anaerobe detection (Butler-Wu and Cookson, 2013). Moreover, MC-ID has confining capabilities in identifying bacteria at the species level. The assay relies on the fragment size profiles of the interspace region between 16S and 23S rDNA for species identification. This limits the ability to discern species that exhibit similarities in length in this region, rendering them indistinguishable without sequencing or other forms of confirmation. Although MC-ID has the potential to detect almost any bacteria, it is limited by the extent of its database. The database will be updated accordingly as larger studies, like the presently described, are performed. MC-ID also lacks the capability to detect fungal species or provide antimicrobial resistance testing.

For this study, only a total white blood cell count was available. Although this parameter is not the main hallmark for BP, leukocyte levels are used to guide clinicians’ diagnosis in similar ways as PMN counts (Li et al., 2022; Kunin et al., 2023). Unfortunately, the waiver filed by the Medical Ethics Committee only allowed the use of SOC results without informed consent. It did not permit us to access any other data from the medical records, such as clinical outcomes, Gram stain, ASTs, antibiotic administration or comorbidities, which could have improved the assessment of the impact of our findings.

In summary, the results presented in this study highlight that MC-ID significantly improved BP detection over SOC by enhancing species identification, especially for difficult to culture organisms. The results of MC-ID align with the leukocyte levels, indicating that MC-ID could guide BP diagnosis. Nevertheless, further optimization is needed to improve sensitivity and enable sequencing-free species resolution for routine diagnostics use. This study did not assess the impact of MC-ID outcomes to guide BP diagnosis, though its potential for same-day results warrants future evaluation. These findings represent meaningful progress in BP care and antimicrobial stewardship.

## Data Availability

The original contributions presented in the study are included in the article and **Supplementary Material**, further inquiries can be directed to the corresponding author.
