# Correction: Diagnostic performance of broad-range PCR in bacterial peritonitis

**DOI:** 10.3389/fcimb.2025.1744166

**Published:** 2025-11-24

**Authors:** María Miguélez Sánchez, Lauren Remijas, Martine P. Bos, Tom Lancee, Robin van Houdt, Andries E. Budding

**Affiliations:** 1 inBiome B. V., Amsterdam, Netherlands; 2 Medical Microbiology and Infection Control, Amsterdam UMC Locatie VUmc, Amsterdam, Netherlands

**Keywords:** peritonitis, molecular diagnosis, broad range PCR assay, ascites fluid infection, PMN (polymorphonuclear leucocyte), molecular diagnosis and epidemiology of enteric infections

The abstract section missing from the final manuscript. This has been corrected to read:

“**Objective:** Bacterial peritonitis (BP) is a serious complication commonly associated with cirrhosis and ascites, often leading to high mortality rates. Although these effects could be reduced with timely and appropriate antibiotics, traditional BP diagnosis relies on culture, often delaying targeted treatment. Therefore, the use of fast molecular assays holds the potential to enhance laboratory diagnosis. In this study, we assessed the diagnostic performance of Molecular Culture ID, a broad PCR-based assay targeting the 16S-23S interspace rDNA region in the scope of BP diagnosis.

**Methods:** The residual material from 247 peritoneal fluid samples submitted for routine diagnostics was analyzed using Molecular Culture ID and compared alongside the standard of care (SOC) results.

**Results:** Sample positivity and species identification outcomes of Molecular Culture ID were compared to those of SOC. Molecular Culture ID yielded 1.6x more positive samples than SOC. Percent positive agreement (PPA) between Molecular Culture ID and SOC at the sample level was 90.1% (IC 95%, 81.0% to 95.1%), and negative percent agreement (NPA) was 70.5% (IC 95%, 63.3% to 76.7%). At the species level, the PPA was 75.2% (95% CI 67.2% to 81.8%). Molecular Culture ID yielded 289 extra bacterial identifications, mainly anaerobic species. High leukocyte counts, indicative of infection, were concordant with Molecular Culture ID positivity.

**Conclusion:** Molecular Culture ID demonstrated enhanced BP diagnostic capabilities compared to SOC, with higher positivity rates, more comprehensive species identification for difficult to culture species and a high correlation with leukocyte counts.”

The original version of this article has been updated.

