# Supplementary material for: Diagnostic performance of broad-range PCR in bacterial peritonitis
Source: Front Cell Infect Microbiol. 2025 Oct 1;15:1645965. doi: 10.3389/fcimb.2025.1645965 (PMC12521200; doi:10.3389/fcimb.2025.1645965)
Supplement: Supplementary file 1 [file DataSheet1.pdf]

SUPPLEMENTARY MATERIAL

Sample number	SOC result	SOC load	MC-ID result	MC-ID load
Sample 1	*Cutibacterium acnes*	0,1	negative	
Sample 2	*Enterococcus faecium*	0,1	negative	
Sample 3	*Escherichia coli*	0,1	negative	
Sample 4	*Staphylococcus capitis*	0,1	negative	
Sample 5	*Staphylococcus aureus*	0,1	negative	
Sample 6	*Enterococcus faecium*	0,1		
			Escherichia coli/Shigella spp.	H
			Sarcina ventriculi	H
Sample 7	*Staphylococcus warneri*	0,1		
			Alloiococcus otitis	H
			Cutibacterium acnes	H
			Staphylococcus capitis	H
Sample 8	*Enterococcus gallinarum*	0,1		
	Escherichia coli	3	*Escherichia coli/Shigella* spp.	H
			Bacteroides vulgatus	H
			Bacteroides cacae	M
			Bacteroides zoogleoformans / Barnesiella merdipullorum	M
Sample 9	*Staphylococcus aureus*	0,1		
	Escherichia coli	1	*Escherichia coli/Shigella* spp.	M
	Proteus vulgaris	1	*Proteus* sp.	L
Sample 10	*Citrobacter koseri*	0,1		
	Neisseria subflava	0,1		
	Enterococcus faecalis	2	*Enterococcus faecalis*	M
	Streptococcus mitis	1	*Streptococcus pneumoniae/mitis* group	M
			Grandulicatella adiacens	H
			Streptococcus cristatus/criceti	L
Sample 11	*Streptococcus parasanguinis*	0,1		
	Streptococcus salivarus	1		
			Turicibacter bilis	H
			Clostridium butyricum	M
			Enterococcus gallinarum	M
			Romboutsia ilealis	M
Sample 12	*Staphylococcus warneri*	1	negative	
	Staphylococcus pasteuri	1		
Sample 13	*Staphylococcus haemolyticus*	1	negative	

Sample 14	*Staphylococcus aureus*	1		
			Klebsiella pneumoniae / Enterobacter cloacae complex	H
Sample 15	*Morganella morganii*	1		
	Enterococcus faecalis	1	*Enterococcus faecalis*	L
	Enterococcus faecium	1	*Enterococcus faecium*	H
			Citrobacter freundii complex	H
			Streptococcus bovis group/*Streptococcus intermedius*	H
Sample 16	*Enterococcus faecalis*	1		
	Enterococcus faecium	2	*Enterococcus faecium*	M
			Clostridiales sp.	M
			Suterella wadsworthensis	H
Sample 17	*Staphylococcus aureus*	1		
	Enterococcus faecalis	1	*Enterococcus faecalis*	H
	Enterobacter cloacae	2	*Klebsiella pneumoniae / Enterobacter cloacae* complex	H
			Dyadobacter spp.	H
Sample 18	*Enterococcus faecium*	1		
			Bacteroides dorei	H
			Haemophilus haemolyticus	H
			Prevotella sp.	M
			Streptococcus bovis group/*Streptococcus intermedius*	M
			Unknown Bacteroidetes	M
			Alistipes group	L
			Streptococcus pneumoniae/mitis group	L
Sample 19	*Corynebacterium tuberculostearicum*	2		
	Staphylococcus epidermidis	2	*Staphylococcus epidermidis*	H
	Escherichia coli	2	*Escherichia coli/Shigella* spp.	H
	Enterococcus faecium	2	*Enterococcus faecium*	M
			Enterococcus faecalis	M
Sample 20	*Acinetobacter sp.*	2		
	Bacillus species	2		
	Klebsiella oxytoca	1	*Klebsiella aerogenes/oxytoca*	M
	Stenotrophomonas maltophilia	3	*Stenotrophomonas maltophilia*	M
			Citrobacter freundii complex	H
			Lactobacillus rhamnosus/zeae/acidophilus	M
			Streptococcus bovis group/*Streptococcus intermedius*	M
			Enterococcus faecalis	L

Sample 21	*Enterococcus faecalis*	2		
	Enterococcus faecium	2		
	Staphylococcus epidermidis	2		
	Escherichia coli	3	*Escherichia coli/Shigella* spp.	M
			Prevotella denticola	H
			Peptostreptococcus anaerobius	H
			Prevotella intermedia	M
			Streptococcus pneumoniae/mitis group	M
Sample 22	*Enterococcus faecalis*	2		
	Klebsiella pneumoniae	3		
	Klebsiella oxytoca	3	*Klebsiella aerogenes/oxytoca*	H
			Alistipes group	H
			Streptococcus bovis group/*Streptococcus intermedius*	H
			Clostridium perfringens	H
			Odoribacter splanchnicus	H
			Streptococcus pneumoniae/mitis group	M
			Bacteroides vulgatus	M
Sample 23	Coagulase Negative *Staphylococci*	3		
	Enterococcus faecium	3	*Enterococcus faecium*	H
			Bacteroides fragilis group	H
Sample 24	*Morganella morganii*	3		
	Enterococcus faecalis	3		
	Escherichia coli	3	*Escherichia coli/Shigella* spp.	H
	Proteus vulgaris	3	*Proteus penneri*	M
			Enterococcus faecalis	H
			Grandulicatella adiacens	H
			Streptococcus pneumoniae/mitis group	M
Sample 25	*Escherichia coli*	3		
	Clostridium perfringens	3	*Clostridium perfringens*	H
	Klebsiella pneumoniae	3	*Klebsiella pneumoniae / Enterobacter cloacae* complex	H
	Fecal microbiota	3	*Bacteroides zoogleoformans / Barnesiella merdipullorum*	M
			Streptococcus bovis group/*Streptococcus intermedius*	H
			Bacteroides fragilis group	H
			Citrobacter koseri/farmeri	M
			Streptococcus pneumoniae/mitis group	M
Sample 26	*Enterococcus faecalis*	3		
	Enterococcus faecium	3	*Enterococcus faecium*	H
	Streptococcus anginosus	3	*Streptococcus anginosus/intermedius*	H

Citrobacter freundii	3	*Citrobacter freundii* complex	M
Enterobacter cloacae	3	*Klebsiella pneumoniae / Enterobacter cloacae* complex	L
		Bacteroides fragilis group	H
		Odoribacter splanchnicus	H
		Bacteroides dorei	M
		Gemella morbillorum/haemolysans	M
		Suterella wadsworthensis	M

Table S1. Summary of samples with discordant identifications found only in SOC and not in MC-ID (n = 26). The load of MC-ID is represented as high (H), medium (M), low (L).

**Species**	**No. of additional identifications by MC-ID**
**Anaerobes**	**166**
Abiotrophia defectiva	1
Actinomyces sp.*	1
*Akkermansia muciniphila**	2
Alistipes group	18
Alloprevotella tannerae	1
Anaerococcus sp.	3[2]
Bacteroides caccae	1
Bacteroides eggerthii	1
Bacteroides fragilis group	8[2]
*Bacteroides dorei**	12
Bacteroides sp.	12
Bacteroides thetaiotaomicron	2
Bacteroides vulgatus	7[1]
Barnesiella merdipullorum	5[2]
Bulleidia extructa	1
Butyricimonas sp.*	3
Capnocytophaga sputigena	3
Clostridium butyricum	1
Clostridium innocuum	2
Clostridium perfringens	3
Cutibacterium acnes	5
Cutibacterium sp.*	1
Dialister pneumosintes	1[1]
Fusobacterium necrophorum	2[1]
Gemella morbillorum/haemolysans	2
Granulicatella adiacens	2[1]
Lactobacillus (para)gasseri	3
Lactobacillus jensenii	1[1]
Lactobacillus sp.	3
Odoribacter splanchnicus	9
*Paeniclostridium sordellii**	1
Parabacteroides distasonis	2

*Parabacteroides intestinipullorum**	1
Peptostreptococcus anaerobius	2[1]
Prevotella buccae	1
Prevotella denticola	2[1]
Prevotella histicola	1
Prevotella intermedia	3
Prevotella melaninogenica/jejuni	2
Prevotella oralis	2[2]
Prevotella pallens	2[2]
Prevotella sp.	1
*Romboutsia ilealis**	3
Streptococcus constellatus	2[1]
*Sutterella wadsworthensis**	12
*Thermobrachium celere**	2
*Turicibacter bilis**	1
Turicibacter sanguinis	2[1]
*Clostridiales**	2
*Ruminococcus bicirculans**	1
Unknown Bacteroidetes **	5
**Enterobacteriaceae**	**24**
Citrobacter freundii complex	4
Citrobacter sp.	1
Citrobacter koseri/farmeri	1
Citrobacter sedlakii	1
Escherichia coli/Shigella spp.	12
Klebsiella pneumoniae complex / *Enterobacter cloacae* complex	4[1]
Serratia marcescens	1
**Enterococci**	**26**
Enterococcus avium	1
Enterococcus cecorum	4[1]
Enterococcus faecalis	11[1]
Enterococcus faecium	9[2]
Enterococcus gallinarum	1
**Staphylococci**	**10**
Staphylococcus aureus	1
Staphylococcus capitis	2
Staphylococcus epidermidis/Streptococcus sanguinis	5
Staphylococcus haemolyticus	1
Staphylococcus hominis	1
**Streptococci**	**36**
Streptococcus agalactiae	1[1]
Streptococcus anginosus	1
Streptococcus bovis group/Streptococcus intermedius	16[1]
Streptococcus cristatus/criceti	1
Streptococcus mitis	1[1]
Streptococcus pneumoniae/mitis group	15[1]
Streptococcus thermophilus	1[1]

Other Gram-Negative	16
Aggregatibacter sp.*	1
Dyadobacter sp.*	1
Eikenella corrodens	1
Haemophilus haemolyticus	2
Haemophilus parainfluenzeae	1[1]
Lautropia mirabilis	1
Massilia sp.*	1
Neisseria mucosa/sicca	1
Neisseria subflava group	1
Pseudomonas aeruginosa	1[1]
Pseudomonas putida	2
Pseudomonas sp.	1
Ralstonia mannitolilytica	1[1]
Stenotrophomonas maltophilia	1
**Other Gram-Positive**	**8**
Alloiococcus otitis	1
Bacillus smithii	4[1]
*Carnobacterium jeotgali**	1
Pediococcus acidilactici	1[1]
Rothia mucilaginosa/aeria	1
Unknown FAFV **	2
Unknown Proteobacteria **	1
**TOTAL**	**289**

* These species are not part of MC-ID database, although MC-ID detected the presence of bacteria the identification was performed through sequencing.

**These unknown Bacteria were only able to be identified at the phylum level.

Table S2. Summary of extra bacterial identifications by MC-ID. The superscript for MC-ID identifications indicates the number of identifications obtained through sequencing the MC-ID outcome.

Sample number	MC-ID result	MC-ID load
**Patient 1**	*Clostridium perfringens*	H
**Patient 2**	*Prevotella melaninogenica/jejuni*	M
	Haemophilus parainfluenzeae	M
	Streptococcus bovis group/*Streptococcus intermedius*	M
	Streptococcus pneumoniae/mitis group	H
**Patient 3**	*Lactobacillus rhamnosus/zeae/acidophilus*	H
	Streptococcus bovis group/*Streptococcus intermedius*	L
**Patient 4**	*Staphylococcus aureus*	M
**Patient 5**	*Escherichia coli/Shigella* spp.	H
**Patient 6**	*Cutibacterium acnes*	H
**Patient 7**	*Klebsiella pneumoniae* complex / *Enterobacter cloacae* complex	M
**Patient 8**	*Cutibacterium* sp.	M
**Patient 9**	*Alistipes* group	H
	Bacteroides dorei	H
	Bacteroides zoogleoformans / Barnesiella merdipullorum	M
	Odoribacter splanchnicus	M
	Streptococcus constellatus	H
	Sutterella wadsworthensis	L
	Klebsiella aerogenes/oxytoca	H
	Pseudomonas putida	L
**Patient 10**	*Escherichia coli/Shigella* spp.	H
**Patient 11**	*Alistipes group*	M
	Cutibacterium acnes	H
	Streptococcus pneumoniae/mitis group	M
**Patient 12**	*Klebsiella pneumoniae complex / Enterobacter cloacae* complex	H
**Patient 13**	Unknown Bacteroidetes	L
**Patient 14**	*Streptococcus pneumoniae/mitis* group	H
**Patient 15**	*Escherichia coli/Shigella* spp.	H
**Patient 16**	*Enterococcus avium*	M
**Patient 17**	*Klebsiella pneumoniae* complex / *Enterobacter cloacae* complex	H
**Patient 18**	*Paeniclostridium sordellii*	H
	Streptococcus agalactiae	H
**Patient 19**	*Prevotella histicola*	M
	Streptococcus bovis group/Streptococcus intermedius	L
	Streptococcus pneumoniae/mitis group	H
	Actinomyces sp.	H
**Patient 20**	*Parabacteroides distasonis*	M
	Parabacteroides intestinipullorum	L
	Citrobacter freundii complex	M

	Enterococcus faecium	L
	Streptococcus bovis group/*Streptococcus intermedius*	L
**Patient 21**	*Unknown FAFV*	L
**Patient 22**	*Neisseria subflava* group	L
	Gemella morbillorum/haemolysans	M
	Prevotella melaninogenica/jejuni	L
	Prevotella pallens	L
	Streptococcus constellatus	M
	Rothia mucilaginosa/aeria	L
	Streptococcus pneumoniae/mitis group	M
	Aggregatibacter sp.	L
**Patient 23**	*Turicibacter sanguinis*	L
	Streptococcus bovis group/*Streptococcus intermedius*	L
**Patient 24**	*Enterococcus cecorum*	L
**Patient 25**	*Lactobacillus (para)gasseri*	L
	Stenotrophomonas maltophilia	L
	Staphylococcus haemolyticus	L
	Corynebacterium sp.	M
**Patient 26**	*Streptococcus pneumoniae/mitis* group	L
	Unknown FAFV	L
**Patient 27**	*Cutibacterium acnes*	H
	Bacillus smithii	L
	Enterococcus cecorum	L
**Patient 28**	*Cutibacterium acnes*	H
**Patient 29**	*Enterococcus faecium*	L
	Staphylococcus hominis	L
**Patient 30**	*Bacillus smithii*	L

Table S3. Summary results of SOC-negative and MC-ID-positive samples with high to medium leukocyte counts (n =30). The load of MC-ID is represented as high (H), medium (M), low (L).
