# Supplementary material for: Diagnostic performance of broad-range PCR in bacterial peritonitis
Source: Front Cell Infect Microbiol. 2025 Oct 1;15:1645965. doi: 10.3389/fcimb.2025.1645965 (PMC12521200; doi:10.3389/fcimb.2025.1645965)
Supplement: Supplementary file 2 [file DataSheet2.docx]

# **SUPPLEMENTARY MATERIAL**

| **Sample number** | **SOC result** | **SOC load** | | **MC-ID result** | **MC-ID load** | |
| --- | --- | --- | --- | --- | --- | --- |
| Sample 1 | *Cutibacterium acnes* | 0,1 | negative | | |  |
| Sample 2 | *Enterococcus faecium* | 0,1 | negative | | |  |
| Sample 3 | *Escherichia coli* | 0,1 | negative | | |  |
| Sample 4 | *Staphylococcus capitis* | 0,1 | negative | | |  |
| Sample 5 | *Staphylococcus aureus* | 0,1 | negative | | |  |
| Sample 6 | *Enterococcus faecium* | 0,1 |  | | |  |
|  |  |  | *Escherichia coli/Shigella* spp. | | | H |
|  |  |  | *Sarcina ventriculi* | | | H |
| Sample 7 | *Staphylococcus warneri* | 0,1 |  | | |  |
|  |  |  | *Alloiococcus otitis* | | | H |
|  |  |  | *Cutibacterium acnes* | | | H |
|  |  |  | *Staphylococcus capitis* | | | H |
| Sample 8 | *Enterococcus gallinarum* | 0,1 |  | | |  |
|  | *Escherichia coli* | 3 | *Escherichia coli/Shigella* spp. | | | H |
|  |  |  | *Bacteroides vulgatus* | | | H |
|  |  |  | *Bacteroides cacae* | | | M |
|  |  |  | *Bacteroides zoogleoformans / Barnesiella merdipullorum* | | | M |
| Sample 9 | *Staphylococcus aureus* | 0,1 |  | | |  |
|  | *Escherichia coli* | 1 | *Escherichia coli/Shigella* spp. | | | M |
|  | *Proteus vulgaris* | 1 | *Proteus* sp. | | | L |
| Sample 10 | *Citrobacter koseri* | 0,1 |  | | |  |
|  | *Neisseria subflava* | 0,1 |  | | |  |
|  | *Enterococcus faecalis* | 2 | *Enterococcus faecalis* | | | M |
|  | *Streptococcus mitis* | 1 | *Streptococcus pneumoniae/mitis* group | | | M |
|  |  |  | *Grandulicatella adiacens* | | | H |
|  |  |  | *Streptococcus cristatus/criceti* | | | L |
| Sample 11 | *Streptococcus parasanguinis* | 0,1 |  | | |  |
|  | *Streptococcus salivarus* | 1 |  | | |  |
|  |  |  | *Turicibacter bilis* | | | H |
|  |  |  | *Clostridium butyricum* | | | M |
|  |  |  | *Enterococcus gallinarum* | | | M |
|  |  |  | *Romboutsia ilealis* | | | M |
| Sample 12 | *Staphylococcus warneri* | 1 | negative | | |  |
|  | *Staphylococcus pasteuri* | 1 |  | | |  |
| Sample 13 | *Staphylococcus haemolyticus* | 1 | negative | | |  |
| Sample 14 | *Staphylococcus aureus* | 1 |  | | |  |
|  |  |  | *Klebsiella pneumoniae / Enterobacter cloacae* complex | | | H |
| Sample 15 | *Morganella morganii* | 1 |  | | |  |
|  | *Enterococcus faecalis* | 1 | *Enterococcus faecalis* | | | L |
|  | *Enterococcus faecium* | 1 | *Enterococcus faecium* | | | H |
|  |  |  | *Citrobacter freundii* complex | | | H |
|  |  |  | *Streptococcus bovis* group*/Streptococcus intermedius* | | | H |
| Sample 16 | *Enterococcus faecalis* | 1 |  | | |  |
|  | *Enterococcus faecium* | 2 | *Enterococcus faecium* | | | M |
|  |  |  | *Clostridiales* sp. | | | M |
|  |  |  | *Suterella wadsworthensis* | | | H |
| Sample 17 | *Staphylococcus aureus* | 1 |  | | |  |
|  | *Enterococcus faecalis* | 1 | *Enterococcus faecalis* | | | H |
|  | *Enterobacter cloacae* | 2 | *Klebsiella pneumoniae / Enterobacter cloacae* complex | | | H |
|  |  |  | *Dyadobacter* spp. | | | H |
| Sample 18 | *Enterococcus faecium* | 1 |  | | |  |
|  |  |  | *Bacteroides dorei* | | | H |
|  |  |  | *Haemophilus haemolyticus* | | | H |
|  |  |  | *Prevotella* sp. | | | M |
|  |  |  | *Streptococcus bovis* group*/Streptococcus intermedius* | | | M |
|  |  |  | Unknown Bacteroidetes | | | M |
|  |  |  | *Alistipes group* | | | L |
|  |  |  | *Streptococcus pneumoniae/mitis* group | | | L |
| Sample 19 | *Corynebacterium tuberculostearicum* | 2 |  | | |  |
|  | *Staphylococcus epidermidis* | 2 | *Staphylococcus epidermidis* | | | H |
|  | *Escherichia coli* | 2 | *Escherichia coli/Shigella* spp. | | | H |
|  | *Enterococcus faecium* | 2 | *Enterococcus faecium* | | | M |
|  |  |  | *Enterococcus faecalis* | | | M |
| Sample 20 | *Acinetobacter sp.* | 2 |  | | |  |
|  | *Bacillus species* | 2 |  | | |  |
|  | *Klebsiella oxytoca* | 1 | *Klebsiella aerogenes/oxytoca* | | | M |
|  | *Stenotrophomonas maltophilia* | 3 | *Stenotrophomonas maltophilia* | | | M |
|  |  |  | *Citrobacter freundii* complex | | | H |
|  |  |  | *Lactobacillus rhamnosus/zeae/acidophilus* | | | M |
|  |  |  | *Streptococcus bovis* group*/Streptococcus intermedius* | | | M |
|  |  |  | *Enterococcus faecalis* | | | L |
| Sample 21 | *Enterococcus faecalis* | 2 |  | | |  |
|  | *Enterococcus faecium* | 2 |  | | |  |
|  | *Staphylococcus epidermidis* | 2 |  | | |  |
|  | *Escherichia coli* | 3 | *Escherichia coli/Shigella* spp. | | | M |
|  |  |  | *Prevotella denticola* | | | H |
|  |  |  | *Peptostreptococcus anaerobius* | | | H |
|  |  |  | *Prevotella intermedia* | | | M |
|  |  |  | *Streptococcus pneumoniae/mitis* group | | | M |
| Sample 22 | *Enterococcus faecalis* | 2 |  | | |  |
|  | *Klebsiella pneumoniae* | 3 |  | | |  |
|  | *Klebsiella oxytoca* | 3 | *Klebsiella aerogenes/oxytoca* | | | H |
|  |  |  | *Alistipes* group | | | H |
|  |  |  | *Streptococcus bovis* group*/Streptococcus intermedius* | | | H |
|  |  |  | *Clostridium perfringens* | | | H |
|  |  |  | *Odoribacter splanchnicus* | | | H |
|  |  |  | *Streptococcus pneumoniae/mitis* group | | | M |
|  |  |  | *Bacteroides vulgatus* | | | M |
| Sample 23 | Coagulase Negative *Staphylococci* | 3 |  | | |  |
|  | *Enterococcus faecium* | 3 | *Enterococcus faecium* | | | H |
|  |  |  | *Bacteroides fragilis group* | | | H |
| Sample 24 | *Morganella morganii* | 3 |  | | |  |
|  | *Enterococcus faecalis* | 3 |  | | |  |
|  | *Escherichia coli* | 3 | *Escherichia coli/Shigella* spp. | | | H |
|  | *Proteus vulgaris* | 3 | *Proteus penneri* | | | M |
|  |  |  | *Enterococcus faecalis* | | | H |
|  |  |  | *Grandulicatella adiacens* | | | H |
|  |  |  | *Streptococcus pneumoniae/mitis* group | | | M |
| Sample 25 | *Escherichia coli* | 3 |  | | |  |
|  | *Clostridium perfringens* | 3 | *Clostridium perfringens* | | | H |
|  | *Klebsiella pneumoniae* | 3 | *Klebsiella pneumoniae / Enterobacter cloacae* complex | | | H |
|  | Fecal microbiota | 3 | *Bacteroides zoogleoformans / Barnesiella merdipullorum* | | | M |
|  |  |  | *Streptococcus bovis* group*/Streptococcus intermedius* | | | H |
|  |  |  | *Bacteroides fragilis* group | | | H |
|  |  |  | *Citrobacter koseri/farmeri* | | | M |
|  |  |  | *Streptococcus pneumoniae/mitis* group | | | M |
| Sample 26 | *Enterococcus faecalis* | 3 |  | | |  |
|  | *Enterococcus faecium* | 3 | *Enterococcus faecium* | | | H |
|  | *Streptococcus anginosus* | 3 | *Streptococcus anginosus/intermedius* | | | H |
|  | *Citrobacter freundii* | 3 | *Citrobacter freundii* complex | | | M |
|  | *Enterobacter cloacae* | 3 | *Klebsiella pneumoniae / Enterobacter cloacae* complex | | | L |
|  |  |  | *Bacteroides fragilis* group | | | H |
|  |  |  | *Odoribacter splanchnicus* | | | H |
|  |  |  | *Bacteroides dorei* | | | M |
|  |  |  | *Gemella morbillorum/haemolysans* | | | M |
|  |  |  | *Suterella wadsworthensis* | | | M |

**Table S1.** Summary of samples with discordant identifications found only in SOC and not in MC-ID (n = 26). The load of MC-ID is represented as high (H), medium (M), low (L).

| **Species** | **No. of additional identifications by MC-ID** |
| --- | --- |
| **Anaerobes** | **166** |
| *Abiotrophia defectiva* | 1 |
| *Actinomyces* sp.* | 1 |
| *Akkermansia muciniphila** | 2 |
| *Alistipes* group | 18 |
| *Alloprevotella tannerae* | 1 |
| *Anaerococcus* sp. | 3^2^ |
| *Bacteroides caccae* | 1 |
| *Bacteroides eggerthii* | 1 |
| *Bacteroides fragilis* group | 8^2^ |
| *Bacteroides dorei** | 12 |
| *Bacteroides* sp. | 12 |
| *Bacteroides thetaiotaomicron* | 2 |
| *Bacteroides vulgatus* | 7^1^ |
| *Barnesiella merdipullorum* | 5^2^ |
| *Bulleidia extructa* | 1 |
| *Butyricimonas* sp.* | 3 |
| *Capnocytophaga sputigena* | 3 |
| *Clostridium butyricum* | 1 |
| *Clostridium innocuum* | 2 |
| *Clostridium perfringens* | 3 |
| *Cutibacterium acnes* | 5 |
| *Cutibacterium* sp.* | 1 |
| *Dialister pneumosintes* | 1^1^ |
| *Fusobacterium necrophorum* | 2^1^ |
| *Gemella morbillorum/haemolysans* | 2 |
| *Granulicatella adiacens* | 2^1^ |
| *Lactobacillus (para)gasseri* | 3 |
| *Lactobacillus jensenii* | 1^1^ |
| *Lactobacillus* sp. | 3 |
| *Odoribacter splanchnicus* | 9 |
| *Paeniclostridium sordellii** | 1 |
| *Parabacteroides distasonis* | 2 |
| *Parabacteroides intestinipullorum** | 1 |
| *Peptostreptococcus anaerobius* | 2^1^ |
| *Prevotella buccae* | 1 |
| *Prevotella denticola* | 2^1^ |
| *Prevotella histicola* | 1 |
| *Prevotella intermedia* | 3 |
| *Prevotella melaninogenica/jejuni* | 2 |
| *Prevotella oralis* | 2^2^ |
| *Prevotella pallens* | 2^2^ |
| *Prevotella* sp. | 1 |
| *Romboutsia ilealis** | 3 |
| *Streptococcus constellatus* | 2^1^ |
| *Sutterella wadsworthensis** | 12 |
| *Thermobrachium celere** | 2 |
| *Turicibacter bilis** | 1 |
| *Turicibacter sanguinis* | 2^1^ |
| *Clostridiales** | 2 |
| *Ruminococcus bicirculans** | 1 |
| Unknown Bacteroidetes ** | 5 |
| **Enterobacteriaceae** | **24** |
| *Citrobacter freundii complex* | 4 |
| *Citrobacter* sp. | 1 |
| *Citrobacter koseri/farmeri* | 1 |
| *Citrobacter sedlakii* | 1 |
| *Escherichia coli/Shigella* spp*.* | 12 |
| *Klebsiella pneumoniae* complex */ Enterobacter cloacae* complex | 4^1^ |
| *Serratia marcescens* | 1 |
| **Enterococci** | **26** |
| *Enterococcus avium* | 1 |
| *Enterococcus cecorum* | 4^1^ |
| *Enterococcus faecalis* | 11^1^ |
| *Enterococcus faecium* | 9^2^ |
| *Enterococcus gallinarum* | 1 |
| **Staphylococci** | **10** |
| *Staphylococcus aureus* | 1 |
| *Staphylococcus capitis* | 2 |
| *Staphylococcus epidermidis/Streptococcus sanguinis* | 5 |
| *Staphylococcus haemolyticus* | 1 |
| *Staphylococcus hominis* | 1 |
| **Streptococci** | **36** |
| *Streptococcus agalactiae* | 1^1^ |
| *Streptococcus anginosus* | 1 |
| *Streptococcus bovis group/Streptococcus intermedius* | 16^1^ |
| *Streptococcus cristatus/criceti* | 1 |
| *Streptococcus mitis* | 1^1^ |
| *Streptococcus pneumoniae/mitis* group | 15^1^ |
| *Streptococcus thermophilus* | 1^1^ |
| **Other Gram-Negative** | **16** |
| *Aggregatibacter* sp.*** | 1 |
| *Dyadobacter* sp.* | 1 |
| *Eikenella corrodens* | 1 |
| *Haemophilus haemolyticus* | 2 |
| *Haemophilus parainfluenzeae* | 1^1^ |
| *Lautropia mirabilis* | 1 |
| *Massilia* sp*.** | 1 |
| *Neisseria mucosa/sicca* | 1 |
| *Neisseria subflava* group | 1 |
| *Pseudomonas aeruginosa* | 1^1^ |
| *Pseudomonas putida* | 2 |
| *Pseudomonas* sp. | 1 |
| *Ralstonia mannitolilytica* | 1^1^ |
| *Stenotrophomonas maltophilia* | 1 |
| **Other Gram-Positive** | **8** |
| *Alloiococcus otitis* | 1 |
| *Bacillus smithii* | 4^1^ |
| *Carnobacterium jeotgali** | 1 |
| *Pediococcus acidilactici* | 1^1^ |
| *Rothia mucilaginosa/aeria* | 1 |
|  |  |
| Unknown FAFV ** | 2 |
| Unknown Proteobacteria ** | 1 |
| **TOTAL** | **289** |

* These species are not part of MC-ID database, although MC-ID detected the presence of bacteria the identification was performed through sequencing.

**These unknown Bacteria were only able to be identified at the phylum level.

**Table S2.** Summary of extra bacterial identifications by MC-ID. The superscript for MC-ID identifications indicates the number of identifications obtained through sequencing the MC-ID outcome.

| **Sample number** | **MC-ID result** | **MC-ID load** |
| --- | --- | --- |
| **Patient 1** | *Clostridium perfringens* | H |
| **Patient 2** | *Prevotella melaninogenica/jejuni* | M |
|  | *Haemophilus parainfluenzeae* | M |
|  | *Streptococcus bovis* group*/Streptococcus intermedius* | M |
|  | *Streptococcus pneumoniae/mitis* group | H |
| **Patient 3** | *Lactobacillus rhamnosus/zeae/acidophilus* | H |
|  | *Streptococcus bovis* group*/Streptococcus intermedius* | L |
| **Patient 4** | *Staphylococcus aureus* | M |
| **Patient 5** | *Escherichia coli/Shigella* spp. | H |
| **Patient 6** | *Cutibacterium acnes* | H |
| **Patient 7** | *Klebsiella pneumoniae* complex */ Enterobacter cloacae* complex | M |
| **Patient 8** | *Cutibacterium* sp. | M |
| **Patient 9** | *Alistipes* group | H |
|  | *Bacteroides dorei* | H |
|  | *Bacteroides zoogleoformans / Barnesiella merdipullorum* | M |
|  | *Odoribacter splanchnicus* | M |
|  | *Streptococcus constellatus* | H |
|  | *Sutterella wadsworthensis* | L |
|  | *Klebsiella aerogenes/oxytoca* | H |
|  | *Pseudomonas putida* | L |
| **Patient 10** | *Escherichia coli/Shigella* spp. | H |
| **Patient 11** | *Alistipes group* | M |
|  | *Cutibacterium acnes* | H |
|  | *Streptococcus pneumoniae/mitis* group | M |
| **Patient 12** | *Klebsiella pneumoniae complex / Enterobacter cloacae* complex | H |
| **Patient 13** | Unknown Bacteroidetes | L |
| **Patient 14** | *Streptococcus pneumoniae/mitis* group | H |
| **Patient 15** | *Escherichia coli/Shigella* spp. | H |
| **Patient 16** | *Enterococcus avium* | M |
| **Patient 17** | *Klebsiella pneumoniae* complex */ Enterobacter cloacae* complex | H |
| **Patient 18** | *Paeniclostridium sordellii* | H |
|  | *Streptococcus agalactiae* | H |
| **Patient 19** | *Prevotella histicola* | M |
|  | *Streptococcus bovis group/Streptococcus intermedius* | L |
|  | *Streptococcus pneumoniae/mitis* group | H |
|  | *Actinomyces* sp. | H |
| **Patient 20** | *Parabacteroides distasonis* | M |
|  | *Parabacteroides intestinipullorum* | L |
|  | *Citrobacter freundii* complex | M |
|  | *Enterococcus faecium* | L |
|  | *Streptococcus bovis* group*/Streptococcus intermedius* | L |
| **Patient 21** | *Unknown FAFV* | L |
| **Patient 22** | *Neisseria subflava* group | L |
|  | *Gemella morbillorum/haemolysans* | M |
|  | *Prevotella melaninogenica/jejuni* | L |
|  | *Prevotella pallens* | L |
|  | *Streptococcus constellatus* | M |
|  | *Rothia mucilaginosa/aeria* | L |
|  | *Streptococcus pneumoniae/mitis* group | M |
|  | *Aggregatibacter* sp. | L |
| **Patient 23** | *Turicibacter sanguinis* | L |
|  | *Streptococcus bovis* group*/Streptococcus intermedius* | L |
| **Patient 24** | *Enterococcus cecorum* | L |
| **Patient 25** | *Lactobacillus (para)gasseri* | L |
|  | *Stenotrophomonas maltophilia* | L |
|  | *Staphylococcus haemolyticus* | L |
|  | *Corynebacterium* sp. | M |
| **Patient 26** | *Streptococcus pneumoniae/mitis* group | L |
|  | Unknown FAFV | L |
| **Patient 27** | *Cutibacterium acnes* | H |
|  | *Bacillus smithii* | L |
|  | *Enterococcus cecorum* | L |
| **Patient 28** | *Cutibacterium acnes* | H |
| **Patient 29** | *Enterococcus faecium* | L |
|  | *Staphylococcus hominis* | L |
| **Patient 30** | *Bacillus smithii* | L |

**Table S3.** Summary results of SOC-negative and MC-ID-positive samples with high to medium leukocyte counts (n =30). The load of MC-ID is represented as high (H), medium (M), low (L).
